# Numerical Simulation of the Response of Concrete Structural Elements Containing a Self-Healing Agent

**DOI:** 10.3390/ma15031233

**Published:** 2022-02-07

**Authors:** Todor Zhelyazov

**Affiliations:** Structural Engineering and Composites Laboratory—SEL, Reykjavik University, Menntavegur 1, IS-102 Reykjavik, Iceland; elovar@yahoo.com

**Keywords:** self-healing of cracks, concrete, constitutive relations, damage mechanics, finite element analysis

## Abstract

Self-healing of a crack is a relatively novel technique allowing for the partial recovery of the initial mechanical characteristics of a structural element after some period of exploitation. By a widely accepted convention, self-healing is either autogenous or autonomous. The former is a mechanism inherent for cementitious composites (in particular—concrete), while the latter is an engineered process. Both autogenous and engineered healing have recently been the object of numerous studies. Despite the large amount of research work being carried out, the potential of this technique has not yet been fully realized. The article focuses on the modeling and the finite element simulation of the recovery of the initial material properties resulting from the sealing of cracks. The employed numerical procedure uses a constitutive relation for concrete based on the continuum damage mechanics. It captures both the strain-softening and the inverse process—the crack healing. Finite element simulations of benchmark cases illustrate the effect of self-healing. The numerically obtained constitutive relations for specimens with and without a healing agent are compared.

## 1. Introduction

The self-healing of cracks implies the material properties’ recovery resulting from the crack sealing [1,2]. Generally, self-healing can be a natural (autogenous) or engineered (autonomous) process. Solutions based on self-healing will possibly contribute to extending the life span of concrete structures. The placement of healing agents (such as encapsulated polymers, minerals, or bacteria) in structural elements programs the subsequent initiation of self-healing upon the realization of specific conditions. More precisely, the release of the self-healing agent, when the cracks unseal the capsules, triggers the process. On the other hand, the introduction of mineral additions, crystalline admixtures, superabsorbent, or other polymers stimulate autogenous self-healing [3].

Using environmentally friendly bacteria such as Bacillus pasteurii can replace the commonly used repair materials for concrete (e.g., epoxy systems and acrylic resins), which are environmentally unfriendly [4]. Moreover, the application of epoxies and acrylic resins is reportedly accompanied by cracking and delamination between concrete and the repair material. Also, it should be noted that an intelligent approach to self-healing is required. Thus, for example, calcium ions can be provided either by internal sources in the cement structure or by adding chemicals such as calcium chloride, calcium nitrate, or calcium lactate [5]. However, the utilization of calcium chloride as a calcium source may cause chloride ion attack and consequently degradation of reinforcement bars [6].

Some authors [7,8] attribute the unexpected longevity of bridges and buildings to autogenous healing, acting simultaneously with other mechanisms. The current understanding of self-healing attributes this phenomenon to an interaction between mechanisms such as continuing hydration, dissolution and crystallization, particle clogging, and carbonation. Presumably, due to the continuing hydration, products of strength comparable to that of calcium silicate hydrate (CSH) gel [9] form. Based on results obtained by microstructural analysis [10], the healed cracks contain CSH, ettringite, and calcium hydroxide (Ca(OH)_2_).

The partial substitution of cement with fly ash, as well as the addition of crystalline admixture, yields the recovery of both compressive strength and electrical resistivity of concrete up to 94% [11]. According to other sources (see for example [12]), after 30 days of water curing, a damaged specimen containing a high volume of fly ash regained 74% of the lost compressive strength, whereas samples without fly ash—68%. Based on research on the contribution to the healing capacity of crystalline admixture (CA), [13] reports a healing rate of 81–93% for specimens containing CA, whereas the healing rate of specimens without CA was 80–86%. In samples containing CA, cracks of width up to 0.25 mm were healed.

Polymers (polyurethane, superabsorbent polymers (SAP), acrylamide, acrylate, epoxy, poly styrene-divinylbenzene, styrene-butadiene rubber, etc.) improve the rate of the regain in the mechanical properties and contribute to the healing of surface cracks [14].

Reportedly, fibers (either steel or synthetic) in fiber-reinforced concrete control the crack opening what results in ease of application of the self-healing products in terms of adhesion to the crack surface and crack filling. Thus, according to [15], in engineered cementitious composites (ECC) containing synthetic fibers (such as polypropylene (PP), ethylene vinyl alcohol (EVA), and poly-vinyl acrylate (PVA)), the precipitation of calcium carbonate potentially increases due to the presence of synthetic fibers. For concrete specimens containing PVA fibers and SAP, [16] reports a similar effect. Self-healing of ECC samples containing supplementary cementitious material (SCM) after having been exposed to various deterioration triggering processes has been studied by [17]. Damage has been induced in test specimens by exposure to natural weathering and moderate controlled humidity in a laboratory environment. After that, the test specimens remained submerged in water for 90 days to trigger the self-healing mechanisms. The evaluation of the mechanical characteristics showed a regain of stiffness and tensile strength of 60% and 70%, respectively. The presence of water or high humidity appeared as a crucial factor for a rapid and effective healing process. The time required for healing varies in the function of the method employed. Thus, autogenous healing of concrete might continue for up to two years, whereas the healing process based on encapsulated cyanoacrylate is completed in less than a minute [18]. 

Ultra-high-performance concrete (UHPC) and ultra-high-performance fiber-reinforced concrete (UHPFRC) have the potential to exhibit self-healing capacity because of the high binder content and the low water/binder ratio [19,20,21]. Also, at the damage state, both UHPC and UHPFRC develop cracks of small width, thus creating favorable conditions for self-healing, either via water emersion or by using healing promoters [22,23,24,25,26,27,28]. Based on the available experimental data, the following set of variables can be used in the study and evaluation of the self-healing [18]: the initial opening of the crack and the age of cracking; the curing conditions during the healing period and its duration; the presence of sustained loading along the healing period, which results into through-crack stress states; and the repeatability of the healing action and its effectiveness in consequence of successive repeated cracking phenomena, at the same and/or at different locations.

The modeling of the self-healing of concrete is in close relation with the simulation of the concrete failure behavior. The fracture of concrete has been investigated in numerous research works [29,30,31,32,33,34,35,36,37,38,39,40,41,42,43,44]. 

Among other approaches suitable to model the self-healing phenomena, Yang et al. [45] discuss the possible implementation of the phase-field (PF) methods for self-healing of cementitious materials. A number of literature sources report the application of the PF approach for modeling the fracture behavior of concrete (see among others [46,47,48]). However, the modeling of the crack-closure effects in the framework of the PF approach still needs researchers’ attention. According to [45], possible axes of investigation are: (i) the implementation of cohesive elements along the crack path to prevent overlapping of the crack faces and (ii) a contact scheme, recently proposed by [49]. 

The deterministic approach to the analysis is often questionable, considering the high scattering in material properties within structural elements and loading. The stochastic perturbation-based finite element method theoretically addresses this issue [50].

Recent studies extend the finite element method by proposing a new finite element formulation with embedded strong discontinuity [51] or by coupling the finite element method (FEM) with the discrete element method (DEM) [52]. These research works provide intuitive decoupling of two different phenomena: damage accumulation in the initially undamaged materials and initiation and propagation of the macroscopic cracks. 

In Ref. [51], by hypothesis, the total displacement in the numerical domain is the sum of a component, obtained by linear elastic analysis (of the continuum part), and a jump, associated with the embedded strong discontinuity. The discontinuity presumably follows an elastic-damage cohesive crack constitutive relationship [53]. The proposed finite element formulation accounts for the self-healing by adding a term to account for the healed material. The approach accounts for the healing agent circulation in the material continuum by a modified Lucas–Washburn model [54,55,56,57]. The capillary pressure is evaluated by the Young–Laplace equation, defining the dynamic contact angle according to [58].

In [51], by assumption, the shear jump is constant along the discontinuity in each element [59]. Thus, some shear-dominant problems might require mesh refinement to improve accuracy [51]. Equilibrium across the discontinuity and the crack-plane displacements are defined at the element level. Therefore, the lack of continuity across the finite element boundaries should be compensated by adopting the mean values at coincident nodes in the constitutive relations [60]. The formulation of the specialized finite element presumes small rotations—this could restrain its application to certain crack width.

Combining the finite element method (FEM) and the discrete element method (DEM) has drawn considerable research attention [61,62,63,64]. Considering that the FEM operates with macroscopic characteristics of the material within equations defined in the framework of continuum mechanics, including failure criteria in the context of the constitutive equations and the DEM is a widely recognized method for modeling the response of granular matter and non-continuum media, combining these two methods appears suitable for simulating the transition from initially undamaged to cracked materials such as geomaterials and concrete. 

The combined FEM–DEM formulation implies a multi-scale approach. The frictional contact properties of the interacting discrete particles (for the DEM) are defined at the micro-scale, while the material properties for the FEM generally reflect experimental data obtained at the macroscopic scale. The multi-scale approach involves an identification procedure [52] that generally complicates the overall implementation.

Oñate et al. [52] use the combined FEM–DEM approach to model the concrete degradation within a complex fluid–solid interaction (FSI). The approach requires the generation of a finite element mesh in the computational domain. Damage accumulation in the continuum (using a finite element analysis) is simulated element by element, employing a standard isotropic damage model along with a Mohr–Coulomb failure criterion. Finite elements exceeding a predefined damage threshold are removed from the mesh and replaced by a set of particles modeled with the DEM. These particles overlap the removed finite elements node by node. DEM particles are placed to a maximum close distance, avoiding the initial indentation. The contact interaction forces computed using DEM are transferred to the FE model. 

Both aforementioned approaches imply complicated numerical procedures accounting for crack initiation and propagation in the continuum. On the other hand, the complication affects only a part of the initial computational domain. 

The article focuses on the numerical simulation of the opening and reclosure of cracks, to model the material properties degradation, due to the former mechanism and the material properties recovery attributed to the latter.

A simplified solution scheme using the finite element method only is applied. Using different methods for different phenomena might be intuitive and consistent. On the other hand, continuum mechanics (specifically, the continuum damage mechanics) describes the microscope manifestation of mechanisms that take place at the microscopic scales. The employed damage model slightly differs from the standard damage model incorporated in the advanced numerical simulations referenced above. The numerical algorithm uses an original procedure that integrates the damage variable into the material constitutive relation. Generally, the concrete fracture is described either by using fracture mechanics or in the context of damage mechanics. Damage mechanics is the preferred option in this study since it allows for modeling the damage accumulation and crack propagation starting with homogeneous isotropic material. Unlike the fracture mechanics approach, there is no need to pre-establish defects in the studied specimen to initiate macroscopic cracking. The present article develops and summarizes some ideas discussed in previously published contributions [65,66].

The underlying hypotheses of the reported study presume that mechanical loading triggers the crack opening while the regain in the mechanical properties results from engineered self-healing. Continuum damage mechanics allows for the modeling of the degradation of the initial mechanical properties via the introduction of a damage variable into the material stress–strain relationship. The damage variable is regarded as an operator acting on the elasticity tensor. Thus, damage variable increase models the damage accumulation in the material, and a critical value of the damage variable corresponds to macroscopic crack initiation in the specified location. Vice versa, the sealing of cracks results in a decrease in the damage variable and a partial regain in the initial mechanical properties. Finite element simulations of some benchmark examples illustrate the self-healing mechanism. Modeled specimens are damaged to introduce multiple cracking patterns (i.e., the load is applied to generate some distributed damage). Then, at a specific moment of the loading history, the activation of the autonomous self-healing leads to a sealing of the formed macroscopic cracks. This mechanism results in a partial regain of the initial material properties. 

## 2. Materials and Methods

The focus of the article is on the finite element analysis of concrete specimens containing a self-healing agent. Within the assumed approach, the material response of concrete is modeled via a coupling between elasticity and damage [33,67].

(1) $$\sigma_{\mathrm{ij}}=\frac{\nu }{\left(1+\nu \right)\left(1-2\nu \right)}E\left(1-D\right)\varepsilon_{\mathrm{kk}}\delta_{\mathrm{ij}}+\frac{1}{\left(1+\nu \right)}E\left(1-D\right)\varepsilon_{\mathrm{ij}}$$

In Equation (1), $\sigma_{\mathrm{ij}}$ denotes the components of the stress tensor, ν is the Poisson’s ratio, E is Young’s modulus of the material (concrete) before damage occurs, D is the damage variable, $\varepsilon_{\mathrm{kk}}$ is the trace of the strain tensor (k=1, 2, 3), $\delta_{\mathrm{ij}}$ is the Kronecker delta, and $\varepsilon_{\mathrm{ij}}$ are the components of the strain tensor.

The damage variable acts directly on Young’s modulus. Theoretically, it evaluates the mechanical damage induced by the applied load.

(2) $$D=f\left(C_{i},{\;\varepsilon }_{D0},{\;D}_{c},{\;\varepsilon }_{\mathrm{eqv}}\right)$$

As shown in Equation (2), the damage variable depends on a set of material constants $\left(C_{i},{\;\varepsilon }_{D0},{\;D}_{c}\right)$ that should be identified through comparison with experimental data, and a variable constructed from the positive eigenvalues of the strain tensor [68].

(3) $$\varepsilon_{\mathrm{eqv}}=\sqrt{\overset{3}{\underset{j=1}{\sum }}\langle \varepsilon_{j}\rangle^{2}}$$

The constant $\varepsilon_{D0}$, (also referred to as damage threshold) controls the transition between the elastic response and the inelastic behavior. The constants C_i_, one set of two constants for tension, and another for compression control the shape of the numerically obtained stress–strain relationship. In Equation (3),

(4) $$\langle \varepsilon_{j}\rangle =\frac{\varepsilon_{j}+\left|\varepsilon_{j}\right|}{2}$$

Damage accumulates provided the variable defined in Equation (3) becomes larger than another model parameter, the damage threshold:

(5) $$\frac{\mathrm{dD}}{\mathrm{dt}}\;\{\begin{array}{c} \begin{array}{ccc} =0 & \mathrm{if} & \varepsilon_{\mathrm{eqv}}\leq \varepsilon_{D0} \end{array}\\ \begin{array}{ccc} >0 & \mathrm{if} & \varepsilon_{\mathrm{eqv}}>\varepsilon_{D0} \end{array} \end{array}$$

Reaching the critical value of the damage variable at a specific location in the continuum $\left(D=D_{c}\right)$ means a macroscopic crack initiation or deactivation of the corresponding finite element. The identification of the material constants is performed through curve fitting. For example, one input parameter of the identification procedure is the elasticity modulus of the undamaged material. Material constants are then varied to match the other material characteristics (such as the concrete compressive or tensile strength). In other words, the stress–strain relationship obtained in the numerical simulation of a characterization test should match, as closest as possible, the experimental one.

**Figure 1:**
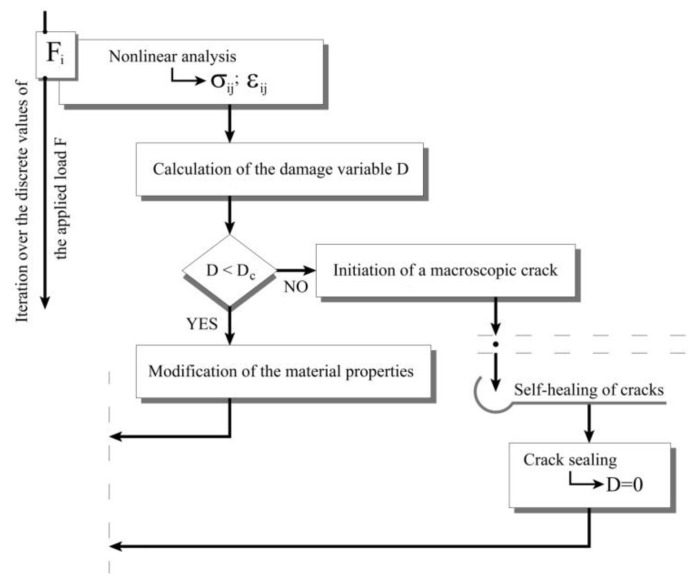
Algorithm for the implementation of the material model taking into account the healing.

The activation of the self-healing process leads to the sealing of the newly formed cracks and regaining the initial rigidity in some regions. Therefore, self-healing is modeled by setting the damage variable equal to zero and the Young modulus—equal to its initial value. At the same time, some level of damage remains in regions where the damage variable has not yet reached its critical value. Figure 1 shows a flowchart of the employed numerical procedure.

Standard compression and tension by flexure tests, as well as a torsion test on concrete specimens, are simulated. Simulations include a preloading phase without reaching the failure load of the specimen. The preloading phase induces some damaged state in the specimen. Also, in some regions, the critical value of the damage variable is reached (i.e., macroscopic cracks are initiated), and the self-healing process is launched (e.g., the healing agent is released from capsules intersected by cracks). The responses of the specimens are obtained by finite element analysis in terms of stress–strain relationships.

The evaluation of the self-healing effectiveness employs an assessment of the recovery of the relevant mechanical properties [18]. Concrete specimens (loaded in compression) exhibit an initial elastic phase. Stress then increases with a decreasing rate, with the increase of the applied quasi-static load up to a maximum value, because of the strain-softening effect. After reaching a specific maximum value, the stress starts to decrease. The mechanical load provokes damage accumulation followed by the initiation of macroscopic crack. By hypothesis, crack initiation (associated with the critical value of the damage variable) triggers autonomous self-healing. The released healing agent seal opened cracks, recovering thus previously degraded stiffness. The stress developed in concrete starts to increase again with the increase of the applied load. In the loading history, including healing, two parameters are to be retained: (i) the maximum stress developed in the specimen during the first loading of the undamaged material (e.g., the compressive strength for concrete loaded in compression) and (ii) the maximum stress developed after the healing. The assessment of the recovery of the material properties consists of a comparison of these two parameters.

## 3. Results Obtained by Finite Element Modeling

The general-purpose finite element code ANSYS is employed for the numerical simulations. A compression test on a cylindrical concrete specimen, a tension by flexure test on a prismatic concrete specimen, and a torsion test on a cylindrical concrete specimen are simulated. Standard geometries are used for the simulations of the compression and the tension-by-flexure tests.

### 3.1. Compression Test

**Figure 2:**
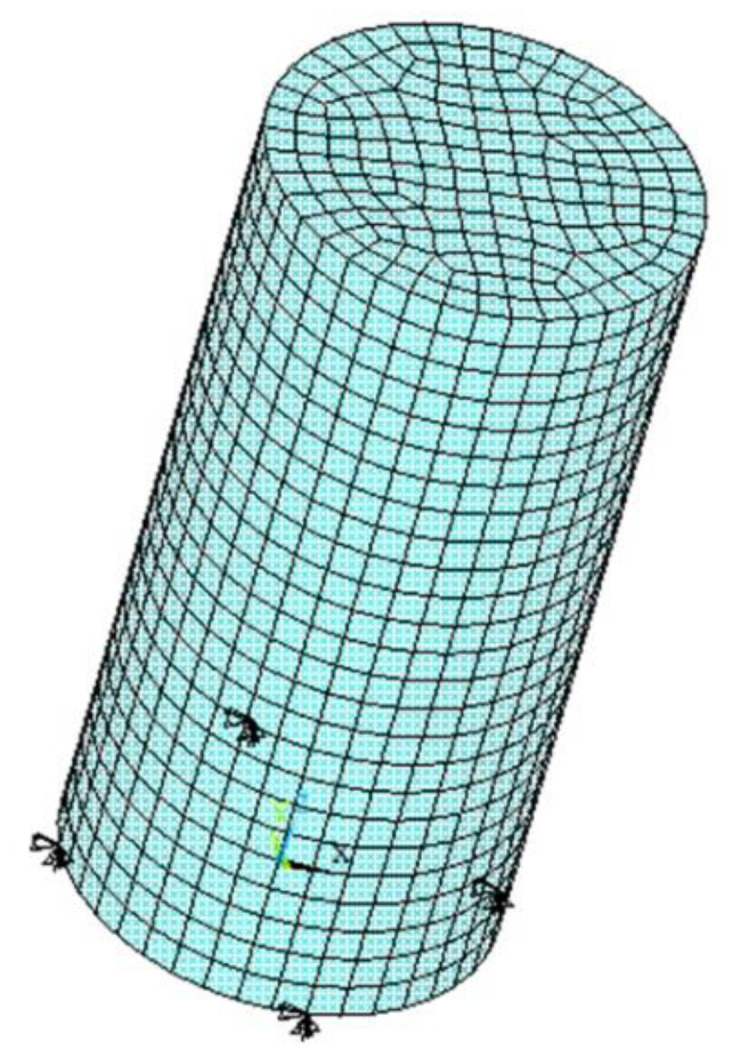
Finite element model built for the compression test.

The compressive strength of the concrete is measured on cylinders 150 mm in diameter and 300 mm in height, in accordance with ISO 1920-3 [69]. Figure 2 displays the finite element model built. The generated finite element mesh contains a total of 4050 finite elements SOLID185. SOLID185 is a 3-D finite element defined by eight nodes, having three degrees of freedom at each node (specifically, translations in the nodal x-, y-, and z-directions). Nodes on the bottom surface (z = 0) are restrained in the z-direction. Additionally, zero displacements in the x- and y-directions are applied to four circumferential nodes on the bottom surface. In the framework of a displacement-controlled simulation, vertical displacements are incrementally applied to top nodes (z = 300 mm).

**Figure 3:**
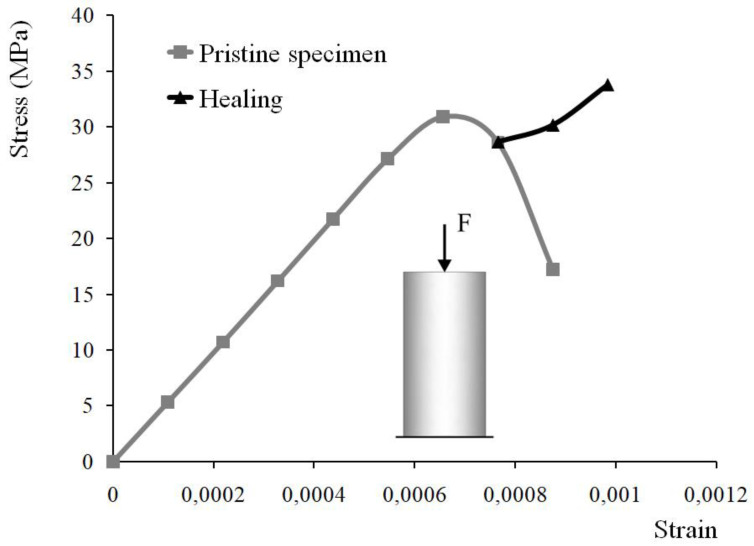
Stress–strain response of a standard cylindrical concrete specimen loaded in compression and then subjected to a self-healing of cracks. The numerically obtained stress–strain relationship for the pristine specimen is shown in grey and the action of the healing agent-in black.

Figure 3 shows results obtained by finite element analysis. As expected, the stress increases with the increase of the applied displacement, and upon reaching the compressive strength, starts to decrease (the grey line in Figure 3). The concrete specimen is loaded in compression without reaching the value of the imposed displacement corresponding to failure. Despite the accumulated damage, the specimen still has some residual load-carrying capacity. At this point, self-healing is initiated, and the previously formed macroscopic cracks are sealed. On the macroscopic scale, crack sealing corresponds to the axial stress increase with the increase of the applied displacement (the black line in Figure 3). 

### 3.2. Tension by Flexure Test

**Figure 4:**
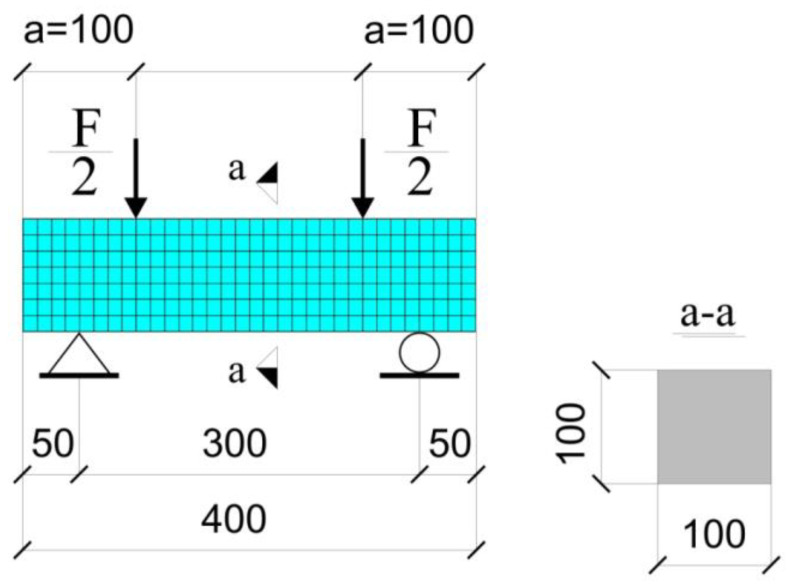
Tension by flexure test: geometry of the modeled specimen and visualization of the generated finite element mesh.

Tension by flexure test commonly performed on a prismatic concrete specimen is simulated. The modeled specimen is 400 mm in length, 300 mm in span, with a rectangular cross-section of 100 mm × 100 mm. The distance between the specimen end and the point load location is 100 mm (denoted by ‘a’ in Figure 4).

According to the beam theory, between the load application points, the specimen is loaded to flexure only. The absence of reinforcement results in the progressive failure of concrete in the tension face, whereas concrete in the compression face still behaves elastically (the elastic domain is commonly assumed up to 30% of the compressive strength). For the considered experimental setup, unstable crack propagation being a fast process, additional time-stepping, with the definition of a smaller time interval, might be required to capture it.

The finite element analysis simulates a displacement-controlled experimental setup with a constant time step for the entire load history. The solid geometry is meshed with 1568 finite elements SOLID185 (Figure 4). To model supports, vertical displacements in the global y-direction of nodes at the corresponding locations are set to zero. Additional restrictions are defined for two nodes to constrain the model. Displacements are incrementally applied to nodes at the load application points (or lines in the three-dimensional model built). 

**Figure 5:**
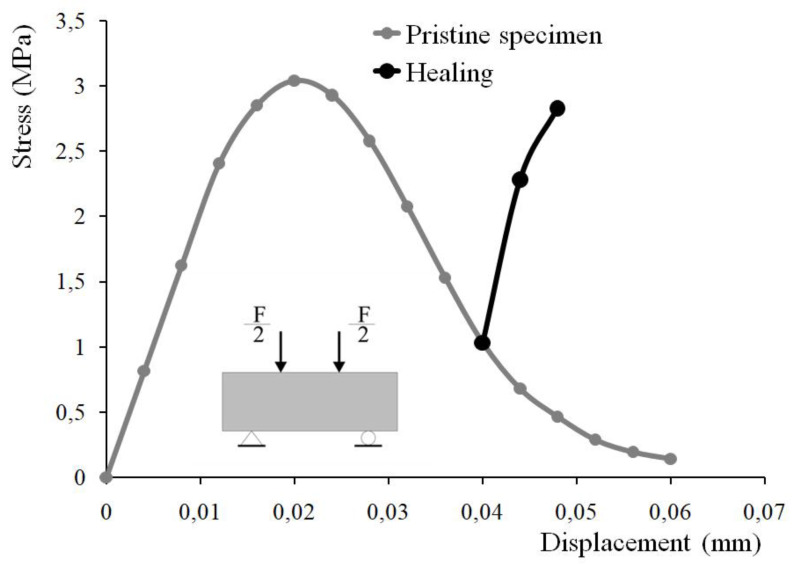
Tension by flexure test: stress–displacement relationship obtained by finite element analysis.

Figure 5 shows the stress–displacement relationship (the grey line) obtained by finite element analysis. Tensile stress in the specimen is calculated based on the beam theory using the gross moment of inertia up to crack initiation. The black line (in Figure 5) reflects the modification of the overall response after the activation of the self-healing mechanism. Cracks’ sealing results in a partial regain of the rigidity and the load-carrying capacity of the damaged specimen. Healing starts after the initiation of macroscopic crack or when the critical value of the damage variable reaches its critical value (in some finite elements). The numerical algorithm implies the deactivation of finite elements with critical damage. Deactivated finite elements do not contribute to the structural element rigidity. The self-healing mechanism reverses this process. With crack reclosure, cracked regions partially restore their initial rigidity.

### 3.3. Torsion Test

**Figure 6:**
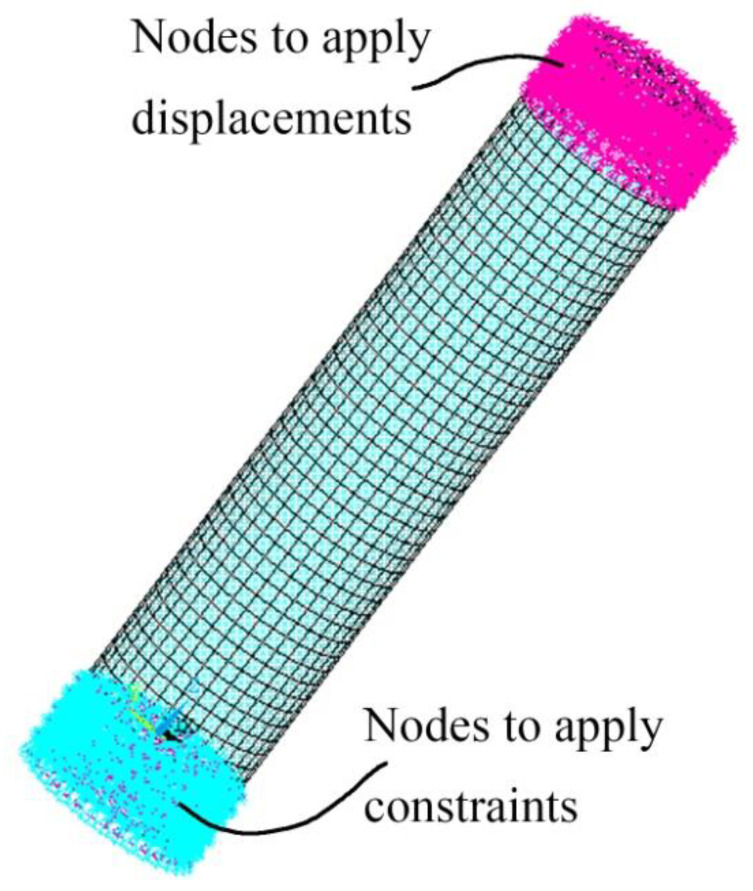
Finite element model built to reproduce the behavior of concrete specimens subjected to torsion: generated finite element mesh and applied boundary conditions.

The finite element simulation reported in this section reproduces the response of a cylindrical concrete specimen subjected to torsion without the ambition to reproduce accurately any experimental setup. Figure 6 displays the finite element model built. It contains a total of 7191 SOLID185 finite elements. Rigid regions are defined where nodes are restrained and where the load is applied to homogenize stress, strain, and damage distributions in the concrete specimen. In the framework of the used cylindrical coordinate system (R, θ, z), the model is constrained by restraining all nodes in the specified region in global z- and tangential θ-directions. Some nodes are also restrained in the radial direction. In another region, displacements are incrementally applied to circumferential nodes, appropriate to model torsional loading.

**Figure 7:**
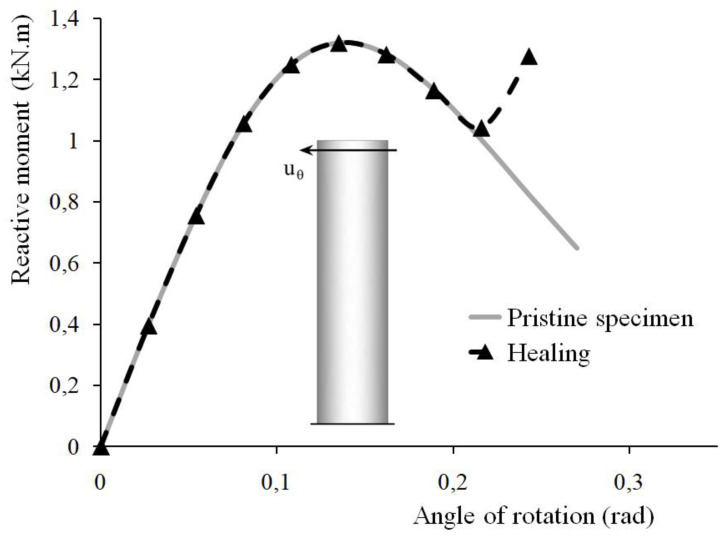
Concrete specimens subjected to torsion: numerically obtained constitutive relations for a specimen containing (dotted black line) and for a specimen without (continuous grey line) healing agent.

Figure 7 presents a comparison between the responses of two concrete specimens loaded in torsion as one of them is subjected to healing after having been damaged. The same load sequence for the incrementally imposed displacements applies for both specimens. The provided comparison between the two relationships (angle of the applied rotation-reactive moment) outlines the positive effect of the self-healing of cracks. As a result of the healing, the apparent regain in the specimen rigidity follows the degradation observed in the pre-loading phase.

## 4. Discussion

Results obtained by finite element simulation have illustrated the effect of the self-healing of cracks. The strength recovery is 109.2%, 93%, and 96.75% for the specimens loaded in compression, flexure, and torsion, respectively. By assumption, the strength recovery is the ratio of the peak values of the stress or moment in the healed and the pristine specimens.

Further implementation of the employed numerical procedure (as a design tool) requires extensive experimental work. The identification of the model parameters, specifically of the critical value of the damage variable, is inherently empirical. Presumably, the aforementioned critical value of the damage variable is directly related to the crack opening and, thereby, the healing process initiation.

The damage-based constitutive relation slightly differs from that implemented in recent research. For example, in [51], the tensile strength of concrete is involved in the damage evolution law, whereas in the present study, the concrete tensile strength is to be matched via the identification of the model constants (please see equation 2). Furthermore, the specimens (for the direct tension test) studied in [51] contain a predefined notch. The specimens modeled herein are initially homogeneous and isotropic. They do not contain any initially defined precursors of cracking.

Very detailed modeling at the mesoscopic scale has been proposed by [70]. To model the fracture at the mesoscale, cohesive elements are inserted at possible failure locations, appropriate constitutive relations and failure criteria are defined. Investigation of some self-healing concrete parameters (such as volume fractions of capsules and aggregates and core-shell thickness of capsules) on the macroscopic response of a specimen loaded in tension. However, the presented results have been limited to the simulation of specimens containing a self-healing agent. Qualitatively, the obtained results reproduce adequately the macroscopic stress–strain relationship for concrete subjected to tension. In contrast, the modeling employed herein accounts implicitly for the action of the healing agent by increasing the value of the damage variable in the modeled compression, tension by flexure, and torsion tests.

Although the self-healing of cracks emerges as a technique capable of enhancing the durability and sustainability of structures, it still possesses some limitations. The major one is that self-healing results in only a partial regaining of the performances of the undamaged material. According to the vast majority of literature sources, the healing rate remains less than 100%. For comparison, alternative techniques for refurbishment, such as strengthened with externally bonded composite materials, provide a net increase in the load-carrying capacity and stiffness. Also, engineered self-healing implies taking actions during construction (such as the self-healing agent encapsulation). In this context, post-strengthening appears to be more flexible for existing structures, especially not equipped for self-healing. On the other hand, when programmed, the engineered self-healing is expected to run as an autonomous process presuming less maintenance and repairs (during the life cycle of a structure).

Despite some shortcomings, the self-healing of cracks remains a field of active research because of the attractive perspectives. Besides autonomous healing, self-healing includes mechanisms inherent for concrete. Accurate quantification of the natural phenomena in the concrete leading to its longevity will contribute to a more efficient design of sustainable structures.

## 5. Conclusions

Numerical simulations of the self-healing process in plain concrete specimens have been presented and discussed. Results obtained by finite element simulations of a compression test, tension by flexure test, and torsion test for healed and pristine specimens of the same geometry have been compared to predict the recovery in strength.

Both material degradation and healing are modeled implicitly in the framework of the continuum damage mechanics. The numerical algorithm integrates into the finite element analysis an original procedure designed to simulate the strain-softening concrete response and extended to account for the effects of healing. The computational procedure uses only the finite element method. It does not include any additional computational scheme to decouple effects due to different mechanisms (i.e., the damage accumulation and crack initiation and propagation). The framework of the continuum damage mechanics is preferred to that of fracture mechanics as it enables modeling of crack propagation and initiation in an initially homogeneous and isotropic material (i.e., there is no need to define initial defect that will subsequently induce macroscopic cracking). 

Numerical results reproduce the response of the pristine specimens well, whereas the estimated recovery in strength for some tests is unexpectedly high, accounting for the empirical data provided by previous (mainly experimental) research works. In this context, a forthcoming experimental campaign can provide additional empirical background for the model validation and the identification of the material constants.

The presented study considers only the healing of specimens subjected to a quasi-static loading. Simulations of the behavior of structural elements under various loading conditions are forthcoming.

## Data Availability

The study employs data available in publicly accessible repositories as well as data available in publicly accessible repositories that do not issue DOIs.
